# Risk factors for postoperative avascular necrosis of the femoral head in children with developmental dysplasia of the hip

**DOI:** 10.3389/fped.2023.1089341

**Published:** 2023-03-06

**Authors:** Ming Yong, Mengqiu Xu, Yue Lou, Gang Lin

**Affiliations:** Department of Orthopedics, Children’s Hospital of Nanjing Medical University, Nanjing, China

**Keywords:** risk factor, avascular necrosis of the femoral head, children, developmental dysplasia of the hip, ANFH

## Abstract

**Aim:**

To investigate factors associated with postoperative avascular necrosis of the femoral head (ANFH) in developmental dysplasia of the hip (DDH) patients, and if or how the associations varied among different subpopulations of age, sex and surgical method.

**Methods:**

Patients with DDH were enrolled between October 31, 2016 and July 15, 2020 in this retrospective cohort study. The average follow-up time was 21.42 ± 10.02 months. The outcome was postoperative ANFH. The main study variables were the DDH classification, Tonnis grade, International Hip Dysplasia Institute (IHDI) classification, and preoperative traction. Multivariate logistic regression was employed to assess the associations between main study variables and postoperative ANFH. Subgroup analysis was carried out based on age at reduction, sex and surgical method. Odds ratio (ORs) and 95% confidence intervals (CIs) were calculated.

**Results:**

A total of 427 children with DDH were included, with 92 (21.55%) in the ANFH group, and 335 (78.45%) in the non-ANFH group. DDH classification was positively correlated with the risk of postoperative ANFH (OR = 4.14, 95% CI, 1.08–15.77, *P *= 0.038). Children with preoperative traction had a significantly decreased risk of postoperative ANFH in contrast to those without preoperative traction (OR = 0.37, 95% CI, 0.22–0.61, *P *< 0.001). Children aged 1–3 years who received preoperative traction has a significantly reduced risk of postoperative ANFH than those who did not receive preoperative traction (OR = 0.28, 95% CI, 0.15–0.51, *P *< 0.001). For children aged >3 years, positive association was found between DDH classification and the risk of postoperative ANFH (OR = 3.75, 95% CI, 1.51–9.31, *P *= 0.004). Girls with a more severe DDH type had a significantly higher risk of postoperative ANFH (OR = 3.80, 95% CI, 1.80–8.02, *P *< 0.001). Receiving preoperative traction was associated with a significantly decreased risk of postoperative ANFH in girls (OR = 0.37, 95% CI, 0.22–0.61, *P *< 0.001). For children undergoing open reduction, DDH classification was positively associated with the risk of postoperative ANFH (OR = 3.01, 95% CI, 1.65–5.50, *P *< 0.001), and those with preoperative traction had a lower risk of postoperative ANFH compared with those without preoperative traction (OR = 0.35, 95% CI, 0.20–0.61, *P *< 0.001).

**Conclusion:**

DDH classification and preoperative traction were associated with the risk of postoperative ANFH, and these associations varied across DDH patients with different ages, sexes and surgical methods.

## Introduction

Developmental dysplasia of the hip (DDH), featured by an abnormal anatomical relationship between the femoral head and the acetabulum, is one of the most common developmental deformities of the lower extremities and one of the primary causes of future osteoarthritis and hip arthroplasty (1, 2). DDH includes a wide range of hip alterations: neonatal instability; acetabular dysplasia; hip subluxation; and true dislocation of the hip. The treatment of DDH generally includes closed reduction and open reduction, which depends on individual patient characteristics (3). Avascular necrosis of the femoral head (ANFH) is the most serious complication and is related to: an excessive abduction of the hip; a force closed reduction when obstacles for reduction are present; a maintained dislocated hip within the harness or spica cast; and a surgical open reduction (4). ANFH is characterized by apoptosis of osteocytes, leading to bone collapse and subsequent involvement of the overlying cartilage, causing flattening of the head surface and the eventual development of secondary osteoarthritis (5, 6).

Risk factors associated with ANFH have been investigated. The degree of hip dislocation (Tonnis classification) (7), traction, age (8), and adductor release (9) were reported as risk factors for ANFH in children with DDH treated by closed reduction. In patients treated with closed reduction younger than 6 months of age, abduction ≥50° was associated with an increased risk of ANFH (8). For the patients older than 10 months, the higher preoperative IHDI grade (IHDI IIII-IV) was a significant risk factor for ANFH (10). Segal et al. (11) showed no significant difference in the occurrence of ANFH with respect to preliminary traction and closed vs. open reduction among children less than 12 months old. According to a previous analysis, children aged <3 years after open reduction exhibited a greater risk of ANFH than those receiving closed reduction (12). A higher rate of ANFH was demonstrated when open reduction was performed in children aged <12 months (13). As indicated above with small sample sizes, the associations between the identified risk factors and ANFH may vary in DDH patients with different ages. Besides, sex and surgical method (closed and open reduction), relating to the risk of ANFH (8, 14), should also be considered when evaluating the risk factors of ANFH, which lacks research at present.

This study aimed to investigate the influencing factors of postoperative ANFH in DDH patients, and if or how the associations varied among different subpopulations of age, sex and surgical method.

## Methods

### Study population

Patients with DDH were enrolled from Nanjing Children's Hospital Affiliated to Nanjing Medical University between October 31, 2016 and July 15, 2020 in this retrospective cohort study. The institutional review board of the hospital approved the study (ethics approval number: 201607025-1). All the patients provided informed consent. The study was performed in accordance with the Declaration of Helsinki. Inclusion criteria were as follows: (1) children aged 0–6 years; (2) patients diagnosed with DDH through medical history, physical examination and x-ray films; (3) patients with complete anteroposterior pelvic radiograph and hip MRI imaging data; (4) patients with no other diseases in the unaffected hip; and (5) patients with complete clinical, laboratory examination and follow-up data. Exclusion criteria: (1) patients with neuromuscular hip dislocation, deformed hip dislocation, and congenital polyarticular contracture hip dislocation; (2) patients with other diseases that affect the development of the hip, such as congenital clubfoot, sequelae of poliomyelitis, and cerebral palsy; (3) patients with serious primary diseases, such as hematopoietic system, and cardiovascular and cerebrovascular diseases, mental patients, and patients with abnormal results of liver and kidney function tests; (4) patients with coagulation dysfunction; (5) patients with preoperative ANFH or a history of preoperative femoral head surgery or heart disease; (6) patients who did not cooperate, could not be contacted after discharge, or refused to be followed up; or (7) patients with unclear surgical methods.

### Follow-up

The patients went to the orthopedic outpatient department for regular review after discharge. The follow-up interval was 1 month for the first 3 months after discharge, 3 months for the next 9 months, and six months after one year. The average follow-up time was 21.42 ± 10.02 months, and the median follow-up time was 20.07 (13.40–27.80) months.

### Data collection

The outcome was postoperative ANFH. The main study variables were the DDH classification (I, II, III), Tonnis grade (I + II, III + IV) (15), International Hip Dysplasia Institute (IHDI) classification (I, II, III, IV) (16), and preoperative traction. DDH was classified into three types: type I was hip dysplasia, type II was subluxation of the hip, and type III was complete dislocation of the hip.

Other variables were also collected, including age at diagnosis (years), age at reduction (years), sex, nationality (Han, other), weight (kg), dislocation side (bilateral, right, left), surgical method (closed reduction, open reduction; closed reduction, Salter pelvic osteotomy + proximal femoral osteotomy, Salter pelvic osteotomy—proximal femoral osteotomy), left acetabular index (degree), right acetabular index (degree), left femoral neck anteversion (FNA), right FNA, left center edge (CE) angle, right CE angle, left collo-diaphyseal angle (CDA), right CDA, ossific nucleus (no, yes), preoperative traction (no, yes), operation time (min), intraoperative blood transfusion (no, yes), adductor muscle release (no, yes), ossification center development (necrotic and deformed; basically normal, small, small and the contralateral femoral head being necrotic and deformed, delayed and absent). An acetabular index was defined as the angle formed by the line between the Y-shaped cartilage centers on both sides and the line between the upper and lower edges of the acetabular surface on the anteroposterior pelvic radiograph, indicating the degree of acetabular development. An FNA was defined as the angle formed by the coronal plane of the femoral neck and the coronal plane of the femoral condyle. A center edge (CE) angle was defined as the angle formed by the line from the center of the femoral head to the outer upper edge of the acetabulum and the perpendicular line through the center of the femoral head in the anteroposterior pelvic radiograph. A CDA was defined as the angle between the longitudinal axis of the femoral shaft and the inner and lower part of the femoral neck axis. A small ossification center was defined as: (1) if the unilateral side was affected, the diameter of the affected side was significantly smaller than that of the healthy side; (2) if both sides were affected, it was smaller than the normal ossification center for the same age. Data were measured by two radiologists and a clinical attending physician in our hospital who had more than five years of clinical work experience, and were qualified after unified training for data measurement. The average values of the measured data from the three persons were taken to reduce measurement errors.

### Statistical analysis

Continuous data with normal distribution were described as the mean ± standard deviation (Mean ± SD), and the t test was used for intergroup comparisons. Continuous data with skewed distribution were expressed as the median and quartile [*M* (*Q*_1_, *Q*_3_)], and comparisons of groups were conducted using the Wilcoxon rank sum test. Categorical data were shown with the number of cases and constituent ratio [*n* (%)], and between-group comparisons were subjected to the *χ*^2^ test or Fisher's exact test. The included patients were divided into groups with and without postoperative ANFH (ANFH group and non-ANFH group). Univariate logistic regression was used to screen for potential confounders associated with postoperative ANFH. Then multivariate logistic regression was employed to assess the associations between main study variables (DDH classification, Tonnis grade, IHDI classification, preoperative traction) and postoperative ANFH. Age at reduction, sex, DDH classification, Tonnis grade, IHDI classification, surgical method, and preoperative traction were adjusted for. For example, when analyzing the effect of age at reduction on postoperative ANFH, we adjusted for sex, DDH classification, Tonnis grade, IHDI classification, surgical method, and preoperative traction. Since surgical open reduction of the femoral head may affect the blood supply of the femoral head (17), we also analyzed the association between surgical methods and postoperative ANFH *via* multivariate analysis. Subgroup analysis was carried out based on age at reduction (<1, 1–3, >3 years), sex and surgical method (closed reduction, open reduction) to further explore whether or how the above associations varied among different subgroups. Odds ratio (ORs) and 95% confidence intervals (CIs) were calculated. SAS v 9.4 (SAS Institute Inc., Cary, NC) was adopted for statistical analysis. All statistical tests were two-tailed, and *P *< 0.05 indicated statistically significant.

## Results

### Patient characteristics

**Figure 1:**
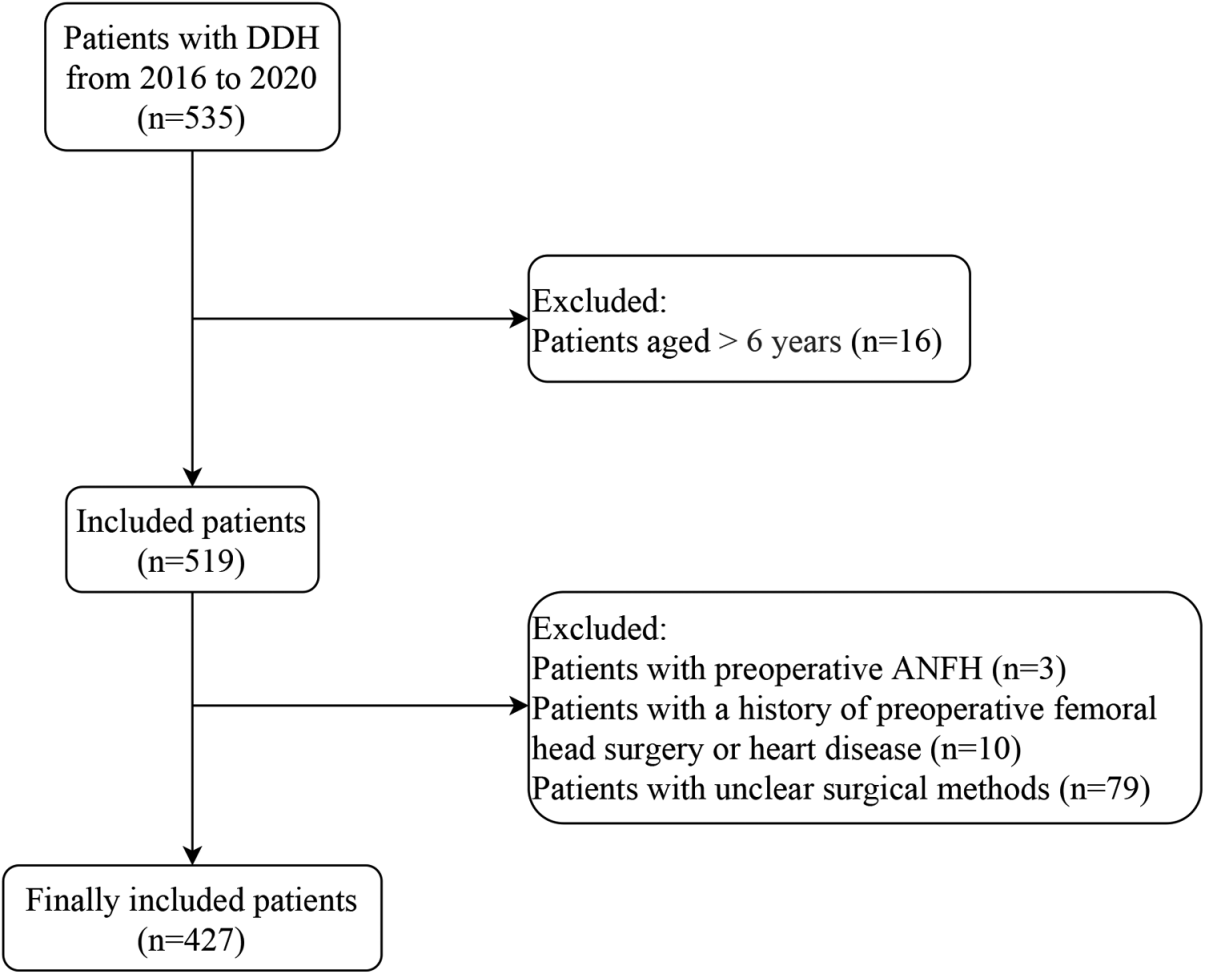
Flow chart of study population screening. DDH, developmental dysplasia of the hip; ANFH, avascular necrosis of the femoral head.

After excluding patients aged >6 years (*n* = 16), and patients with preoperative ANFH (*n* = 3), a history of preoperative femoral head surgery or heart disease (*n* = 10), and unclear surgical methods (*n* = 79), a total of 427 patients with DDH were include in this study, with 92 (21.55%) in the ANFH group, and 335 (78.45%) in the non-ANFH group. Figure 1 presents the screening process of the study population. Age at diagnosis and reduction was 1.25 (1.00, 1.50) and 1.58 (1.30, 2.42) years, respectively. Based on age at reduction, 71 (16.63%) children were <1 year old, 275 (64.40%) were 1–3 years old, and 81 (18.97%) were >3 years old. Thirty-four (7.96%) patients were boys, and 393 (92.04%) were girls; 107 (25.06%) received closed reduction, and 320 (74.94%) received open reduction. The features of the included patients are presented in Table 1. More patients had type-III DDH in the ANFH group vs. non-ANFH group (93.48% vs. 80.60%, *P *= 0.007). The Tonnis grade of the ANFH group was significantly higher than that of the non-ANFH group (*P *= 0.031). Significant differences were also found in the IHDI classification, preoperative traction and surgical method between the two groups (all *P *< 0.05).

**Table 1:** Characteristics of the included patients with DDH.

Variables	Total (n = 427)	ANFH group (n = 92)	Non-ANFH group (n = 335)	Statistics	*P*
Age at diagnosis, years, M (Q_1_, Q_3_)	1.25 (1.00, 1.50)	1.25 (1.00, 1.50)	1.25 (0.83, 1.50)	Z = 0.377	0.706
Age at reduction, years, M (Q_1_, Q_3_)	1.58 (1.30, 2.42)	1.58 (1.33, 2.50)	1.58 (1.25, 2.42)	Z = 0.575	0.566
Age at reduction, years, *n* (%)				*χ*^2^ = 0.591	0.744
<1	71 (16.63)	15 (16.30)	56 (16.72)		
1–3	275 (64.40)	57 (61.96)	218 (65.07)		
>3	81 (18.97)	20 (21.74)	61 (18.21)		
Sex, *n* (%)				χ^2^ = 1.352	0.245
Male	34 (7.96)	10 (10.87)	24 (7.16)		
Female	393 (92.04)	82 (89.13)	311 (92.84)		
Nationality, *n* (%)				–	0.069
Han	422 (98.83)	89 (96.74)	333 (99.40)		
Other	5 (1.17)	3 (3.26)	2 (0.60)		
Weight, kg, Mean ± SD	11.67 ± 3.56	11.69 ± 3.55	11.67 ± 3.57	t = 0.06	0.953
DDH classification, *n* (%)				χ^2^ = 9.806	0.007
I	45 (10.54)	2 (2.17)	43 (12.84)		
II	26 (6.09)	4 (4.35)	22 (6.57)		
III	356 (83.37)	86 (93.48)	270 (80.60)		
Tonnis grade, *n* (%)				χ^2^ = 4.630	0.031
I + II	264 (61.83)	48 (52.17)	216 (64.48)		
III + IV	163 (38.17)	44 (47.83)	119 (35.52)		
IHDI classification, n (%)				χ^2^ = 13.575	0.004
I	45 (10.54)	3 (3.26)	42 (12.54)		
II	134 (31.38)	27 (29.35)	107 (31.94)		
III	169 (39.58)	35 (38.04)	134 (40.00)		
IV	79 (18.50)	27 (29.35)	52 (15.52)		
Dislocation side, n (%)				χ^2^ = 1.157	0.561
Bilateral	125 (29.27)	24 (26.09)	101 (30.15)		
Right	117 (27.40)	29 (31.52)	88 (26.27)		
Left	185 (43.33)	39 (42.39)	146 (43.58)		
Surgical method, n (%)				χ^2^ = 1.212	0.271
Closed reduction	107 (25.06)	19 (20.65)	88 (26.27)		
Open reduction	320 (74.94)	73 (79.35)	247 (73.73)		
Surgical method, n (%)				χ^2^ = 6.350	0.042
Closed reduction	107 (25.06)	19 (20.65)	88 (26.27)		
Salter + proximal femoral osteotomy	168 (39.34)	30 (32.61)	138 (41.19)		
Salter—proximal femoral osteotomy	152 (35.60)	43 (46.74)	109 (32.54)		
Left acetabular index, degree, Mean ± SD	35.82 ± 8.83	36.18 ± 8.49	35.73 ± 8.93	t = 0.44	0.659
Right acetabular index, degree, Mean ± SD	32.60 ± 9.13	33.01 ± 9.05	32.49 ± 9.16	t = 0.48	0.630
Left FNA, degree, M (Q_1_, Q_3_)	26.00 (21.00, 37.00)	28.00 (21.00, 38.00)	26.00 (21.00, 36.00)	Z = 0.770	0.441
Right FNA, degree, M (Q_1_, Q_3_)	24.00 (19.00, 34.00)	27.00 (19.50, 36.00)	24.00 (19.00, 33.00)	Z = 1.478	0.139
Left CE angle, degree, M (Q_1_, Q_3_)	−21.00 (−32.00, 16.00)	−22.00 (−36.50, 15.00)	−19.00 (−31.00, 16.00)	Z = −1.617	0.106
Right CE angle, degree, M (Q_1_, Q_3_)	11.00 (−27.00, 20.00)	11.00 (−26.00, 20.50)	11.00 (−27.00, 20.00)	Z = −0.282	0.778
Left CDA, degree, Mean ± SD	138.99 ± 5.23	138.91 ± 6.93	139.01 ± 4.67	t = −0.12	0.904
Right CDA, degree, Mean ± SD	138.20 ± 5.48	138.35 ± 6.46	138.16 ± 5.19	t = 0.25	0.802
Ossific nucleus, n (%)				χ^2^ = 0.003	0.958
No	55 (12.88)	12 (13.04)	43 (12.84)		
Yes	372 (87.12)	80 (86.96)	292 (87.16)		
Preoperative traction, n (%)				χ^2^ = 8.528	0.003
No	171 (40.05)	49 (53.26)	122 (36.42)		
Yes	256 (59.95)	43 (46.74)	213 (63.58)		
Operation time, min, M (Q_1_, Q_3_)	100.00 (50.00, 157.00)	92.50 (67.50, 180.00)	100.00 (40.00, 155.00)	Z = 0.687	0.492
Intraoperative blood transfusion, n (%)				χ^2^ = 2.081	0.149
No	167 (39.11)	30 (32.61)	137 (40.90)		
Yes	260 (60.89)	62 (67.39)	198 (59.10)		
Adductor muscle release, n (%)				χ^2^ = 0.205	0.650
No	131 (30.68)	30 (32.61)	101 (30.15)		
Yes	296 (69.32)	62 (67.39)	234 (69.85)		
Ossification center development, n (%)				–	<0.001
Necrotic and deformed	1 (0.23)	1 (1.09)	0 (0.00)		
Basically normal	54 (12.65)	0 (0.00)	54 (16.12)		
Small	312 (73.07)	76 (82.61)	236 (70.45)		
Small and the contralateral femoral head being necrotic and deformed	1 (0.23)	0 (0.00)	1 (0.30)		
Delayed and absent	59 (13.82)	15 (16.30)	44 (13.13)		

–: Fisher's exact test.

DDH, developmental dysplasia of the hip; ANFH, avascular necrosis of the femoral head; M, median; SD, standard deviation; DDH, developmental dysplasia of the hip; IHDI, International Hip Dysplasia Institute; FNA, femoral neck anteversion; CE, center edge; CDA, collo−diaphyseal angle.

### Variables associated with postoperative ANFH

According to univariate logistic regression analysis, the DDH classification, Tonnis grade, IHDI classification, and preoperative traction were in significant associations with postoperative ANFH. DDH classification was positively associated with the risk of postoperative ANFH (OR = 2.36, 95% CI, 1.32–4.24, *P *= 0.004). Compared with patients with the Tonnis grades I + II, those with the Tonnis grades III + IV had increased risk of postoperative ANFH (OR = 1.66, 95% CI, 1.04–2.65, *P *= 0.032). The risks of postoperative ANFH were significantly greater in children with the IHDI types II (OR = 3.53, 95% CI, 1.02–12.27, *P *= 0.047), III (OR = 3.66, 95% CI, 1.07–12.50, *P *= 0.039) and IV (OR = 7.27, 95% CI, 2.06–25.63, *P *= 0.002) vs. the IHDI type IV. Receiving preoperative traction was associated with a lower risk of postoperative ANFH (OR = 0.50, 95% CI, 0.32–0.80, *P *= 0.004) (Table 2).

**Table 2:** Variables associated with postoperative ANFH.

Variables	OR (95% CI)	*P*
Age at diagnosis	1.11 (0.84–1.46)	0.468
**Age at reduction**		
<1	Ref	
1–3	0.98 (0.51–1.85)	0.941
>3	1.22 (0.57–2.62)	0.603
**Sex**		
Male	Ref	
Female	0.63 (0.29–1.38)	0.248
**Nationality, n (%)**		
Han	Ref	
Other	5.61 (0.92–34.09)	0.061
Weight	1.01 (0.94–1.07)	0.952
DDH classification	2.36 (1.32–4.24)	0.004
**Tonnis grade**		
I + II	Ref	
III + IV	1.66 (1.04–2.65)	0.032
**IHDI classification**		
I	Ref	
II	3.53 (1.02–12.27)	0.047
III	3.66 (1.07–12.50)	0.039
IV	7.27 (2.06–25.63)	0.002
**Dislocation side**		
Bilateral	Ref	
Right	1.39 (0.75–2.56)	0.295
Left	1.12 (0.64–1.98)	0.687
**Surgical method**		
Closed reduction	Ref	
Open reduction	1.37 (0.78–2.40)	0.272
**Surgical method**		
Closed reduction	Ref	
Salter + proximal femoral osteotomy	1.01 (0.53–1.90)	0.983
Salter—proximal femoral osteotomy	1.83 (0.99–3.36)	0.052
Left acetabular index	1.01 (0.98–1.03)	0.658
Right acetabular index	1.01 (0.98–1.03)	0.629
Left FNA	1.01 (0.99–1.03)	0.321
Right FNA	1.01 (0.99–1.04)	0.174
Left CE angle	0.99 (0.98–0.99)	0.079
Right CE angle	0.99 (0.99–1.01)	0.614
Left CDA	0.99 (0.95–1.04)	0.880
Right CDA	1.01 (0.96–1.05)	0.776
**Ossific nucleus**		
No	Ref	
Yes	0.98 (0.49–1.95)	0.958
**Preoperative traction**		
No	Ref	
Yes	0.50 (0.32–0.80)	0.004
Operation time	0.99 (0.99–0.99)	0.861
**Intraoperative blood transfusion**		
No	Ref	
Yes	1.43 (0.88–2.33)	0.150
**Adductor muscle release**		
No	Ref	
Yes	0.89 (0.54–1.46)	0.651
**Ossification center development**		
Necrotic and deformed	–	0.989
Basically normal	Ref	
Small	–	0.949
Small and the contralateral femoral head being necrotic and deformed	–	1.000
Delayed and absent	–	0.948

ANFH, avascular necrosis of the femoral head; OR, odds ratio; CI, confidence interval; ref: reference; DDH, developmental dysplasia of the hip; IHDI, International Hip Dysplasia Institute; FNA, femoral neck anteversion; CE, center edge; CDA, collo-diaphyseal angle.

### Influencing factors of postoperative ANFH

After multivariate analysis, DDH classification was shown to be in a positive correlation with the risk of postoperative ANFH (OR = 4.14, 95% CI, 1.08–15.77, *P *= 0.038). Children with preoperative traction had a significantly decreased risk of postoperative ANFH in contrast to those without preoperative traction (OR = 0.37, 95% CI, 0.22–0.61, *P *< 0.001). A comparable risk of postoperative ANFH was observed in patients with the Tonnis grades I + II and III + IV (*P *= 0.781). The IHDI type IV related to a similar risk of postoperative ANFH to the IHDI types II, III and IV (all *P *> 0.05) (Table 3).

**Table 3:** Influencing factors of postoperative ANFH.

Variables	OR (95% CI)	*P*
**Age at reduction**		
<1	Ref	
1–3	0.57 (0.17–1.93)	0.363
>3	0.98 (0.23–4.07)	0.973
**Sex**		
Male	Ref	
Female	0.63 (0.27–1.49)	0.295
DDH classification	4.14 (1.08–15.77)	0.038
**Tonnis grade**		
I + II	Ref	
III + IV	0.89 (0.42–1.88)	0.760
**IHDI classification**		
I	Ref	
II	0.66 (0.05–9.52)	0.759
III	0.56 (0.03–9.20)	0.682
IV	1.22 (0.07–22.08)	0.891
**Surgical method**		
Closed reduction	Ref	
Open reduction	2.13 (0.69–6.58)	0.188
**Preoperative traction**		
No	Ref	
Yes	0.37 (0.22–0.61)	<0.001

Age at reduction, sex, DDH classification, Tonnis grade, IHDI classification, surgical method, and preoperative traction were adjusted for. For example, when analyzing the effect of age at reduction on postoperative ANFH, we adjusted for sex, DDH classification, Tonnis grade, IHDI classification, surgical method, and preoperative traction.

ANFH, avascular necrosis of the femoral head; OR, odds ratio; CI, confidence interval; ref: reference; DDH, developmental dysplasia of the hip; IHDI, International Hip Dysplasia Institute.

### Association of DDH classification and preoperative traction with postoperative ANFH in age at reduction subgroups

As illustrated in Table 4, children aged 1–3 years who received preoperative traction has a significantly reduced risk of postoperative ANFH than those who did not receive preoperative traction (OR = 0.28, 95% CI, 0.15–0.51, *P *< 0.001). For children aged >3 years, positive association was found between DDH classification and the risk of postoperative ANFH (OR = 3.75, 95% CI, 1.51–9.31, *P *= 0.004).

**Table 4:** Association of DDH classification and preoperative traction with ANFH in age at reduction subgroups.

Variables	Age at reduction, years					
	<1		1–3		>3	
	OR (95% CI)	*P*	OR (95% CI)	*P*	OR (95% CI)	*P*
DDH classification	–		2.30 (0.94–5.64)	0.069	3.75 (1.51–9.31)	0.004
**Surgical method**						
Closed reduction	Ref		Ref		Ref	
Open reduction	–		1.97 (0.65–6.01)	0.233	–	
**Preoperative traction**						
No	Ref		Ref		Ref	
Yes	0.67 (0.21–2.12)	0.496	0.28 (0.15–0.51)	<0.001	0.85 (0.25–2.88)	0.797

DDH classification, surgical method, and preoperative traction were adjusted for. For example, when analyzing the effect of DDH classification on postoperative ANFH, we adjusted for surgical method and preoperative traction.

ANFH, avascular necrosis of the femoral head; DDH, developmental dysplasia of the hip; OR, odds ratio; CI, confidence interval; ref: reference.

### Association of DDH classification and preoperative traction with postoperative ANFH in sex subgroups

Girls with a more severe DDH type had a significantly higher risk of postoperative ANFH (OR = 3.80, 95% CI, 1.80–8.02, *P *< 0.001). Receiving preoperative traction was associated with a significantly decreased risk of postoperative ANFH in girls (OR = 0.37, 95% CI, 0.22–0.61, *P *< 0.001) (Table 5).

**Table 5:** Association of DDH classification and preoperative traction with postoperative ANFH in sex subgroups.

Variables	Sex			
	Male		Female	
	OR (95% CI)	*P*	OR (95% CI)	*P*
DDH classification	1.15 (0.33–3.98)	0.829	3.80 (1.80–8.02)	<0.001
**Surgical method**				
Closed reduction	Ref		Ref	
Open reduction	–		1.42 (0.78–2.56)	0.252
**Preoperative traction**				
No	Ref		Ref	
Yes	0.71 (0.13–3.99)	0.701	0.37 (0.22–0.61)	<0.001

DDH classification, surgical method, and preoperative traction were adjusted for. For example, when analyzing the effect of DDH classification on postoperative ANFH, we adjusted for surgical method and preoperative traction.

ANFH, avascular necrosis of the femoral head; DDH, developmental dysplasia of the hip; OR, odds ratio; CI, confidence interval; ref, reference.

### Association of DDH classification and preoperative traction with postoperative ANFH in surgical method subgroups

For children undergoing open reduction, DDH classification was positively associated with the risk of postoperative ANFH (OR = 3.01, 95% CI, 1.65–5.50, *P *< 0.001), and those with preoperative traction had a lower risk of postoperative ANFH compared with those without preoperative traction (OR = 0.35, 95% CI, 0.20–0.61, *P *< 0.001) (Table 6).

**Table 6:** Association of DDH classification and preoperative traction with postoperative ANFH in surgical method subgroups.

Variables	Surgical method			
	Open reduction		Closed reduction	
	OR (95% CI)	*P*	OR (95% CI)	*P*
DDH classification	3.01 (1.65–5.50)	<0.001	–	
**Preoperative traction**				
No	Ref		Ref	
Yes	0.35 (0.20–0.61)	<0.001	0.61 (0.22–1.67)	0.333

DDH classification and preoperative traction were adjusted for. For example, when analyzing the effect of DDH classification on postoperative ANFH, we adjusted for preoperative traction.

ANFH, avascular necrosis of the femoral head; DDH, developmental dysplasia of the hip; OR, odds ratio; CI, confidence interval; ref, reference.

## Discussion

The current study first evaluated the associations of DDH classification and preoperative traction with the risk of postoperative ANFH by age at reduction (<1, 1–3, >3 years), sex and surgical method (closed reduction, open reduction). We found that for children aged 1–3 years, preoperative traction was associated with a significantly reduced risk of postoperative ANFH, and for children aged >3 years, DDH classification was positively associated with the risk of postoperative ANFH; among girls, those with a more severe DDH type had a significantly higher risk of postoperative ANFH, and preoperative traction was correlated with a significantly decreased risk of postoperative ANFH; regarding children undergoing open reduction, DDH classification was in a positive association with the risk of postoperative ANFH, and those with preoperative traction had a lower risk of postoperative ANFH than those without. The associations were not significant in other subgroups of age at reduction, sex and surgical method.

Bradley et al. (18) showed 10% overall rate of significant ANFH following closed reduction. In another study, the rate of ANFH was 11%–18% among children of walking age under 18 months old with closed reduction for DDH (9). A meta-analysis showed 73 cases (36%) of ANFH after open reduction and 80 (21%) after closed reduction (12). This study reported an approximately 21.6% rate of ANFH, which considered both closed and open reduction (including Salter pelvic osteotomy with/without proximal femoral osteotomy). At present, evidence on the relationship between DDH classification and the risk of postoperative ANFH is limited. The current study found that DDH classification was positively associated with the risk of postoperative ANFH. When DDH classification was higher, the femoral head was in a high dislocation state before operation, which could result in obvious pathological changes of the hip joint. Complex operation, increased operation time, increased collateral damage, especially the possibility of damage to the blood supply of the femoral head, poor matching of the femoral head and acetabulum, increased pressure of the femoral head and acetabulum after reduction, and sudden changes in the internal mechanical environment of the hip after operation may lead to an increase in the incidence of osteonecrosis. According to a prior meta-analysis by Li et al. (19), preliminary traction does not decrease the incidence of ANFH in DDH treated by closed reduction between 6 and 24 months of age, while our study demonstrated that preoperative traction was associated with a reduced risk of postoperative ANFH. Only one Chinese study on 385 patients was included in the former meta-analysis, and more research is necessitated to validate the association between preoperative traction and postoperative ANFH among patients with DDH. In this study, the traction method was skin traction, the traction direction was the trunk direction, and the hip and knee joints were extended. The feet of the bed were raised to form a 15–30 degree angle between the bed level and the horizontal plane. The applied weight was about 1.5–3 kg (1/7–1/10 of the body weight), and the traction lasted for 2–3 weeks. Preliminary traction may lower the risk of ANFH by reducing the tightness of the hip musculature. Similarly, effective preliminary traction was shown to reduce the incidence of ANFH in patients surgically treated for congenital dislocation of the hip, and the age of patients affected the effectiveness of traction (20).

Age at reduction has been reported to correlate with the occurrence of ANFH (21, 22). Children with developmental dysplasia of the hip, less than 1 year old, can be treated conservatively with closed reduction. The success rate of closed reduction in children aged 1 year and more gradually decreases and the possibility of open reduction increases. The degree of dislocation of the femoral head in children aged 1–3 years is not high, and the pressure of the femoral head is not great after the reduction. Most of them do not need proximal femoral osteotomy. Children over 3 years of age often have high dislocation of the hip joint, large anteversion angle, difficulty in forced reduction, instability of the hip joint, or excessive pressure on the femoral head, leading to necrosis of the femoral head, so proximal femoral osteotomy is often performed during the operation. Salter pelvic osteotomy, the main surgical method used in this study, is usually performed in children aged 6 years and less. Thus, we considered children aged 0–6 years, and conducted subgroup analysis based on age at reduction of <1, 1–3 and >3 years. Given the role of age at reduction, we further found that for children aged 1–3 years, preoperative traction was in a negative association with the risk of postoperative ANFH, and for children aged >3 years, DDH classification was positively associated with the risk of postoperative ANFH. In this study, children aged 18 months and less underwent closed reduction, and children aged more than 18 months underwent open reduction. Salter pelvic osteotomy was performed for those who were more than 18 months old, had a proper ratio of the femoral head and acetabulum, and had an acetabulum index less than 45. All children older than 3 years underwent open reduction. Hence, more studies are needed to investigate the effect of the different surgical methods after 3 years old on the outcome. Appropriate treatments should be applied depending on the age of patients (23). Schur et al. (8) found that the risk of ANFH was affected by sex. According to this paper, among girls, a more severe DDH type was associated with a significantly higher risk of postoperative ANFH, and preoperative traction was correlated with a significantly decreased risk of postoperative ANFH, while no relationships were identified between DDH classification and preoperative traction and the risk of ANFH in boys. Surgical open reduction of the femoral head may affect the blood supply of the femoral head (17). The operation of open reduction is complex, operation time is long, and the collateral injury, especially injury to the blood supply nutrient arteries of the femoral head such as the medial femoral circumflex artery, may negatively affect the blood supply of the femoral head (24). We demonstrated *via* multivariate analysis that closed reduction and open reduction had comparable effects on the incidence of osteonecrosis. For children under 3 years old with DDH, open reduction alone is most likely to be the treatment with the highest incidence rate of ANFH, followed by open reduction combined with osteotomy, and closed reduction is the least likely to cause ANFH (14), suggesting that the association of DDH classification and preoperative traction with the risk of ANFH may vary in DDH patients with closed and open reduction. Then we explored this potential variation across closed and open reduction populations, and demonstrated that for children undergoing open reduction, DDH classification was positively associated with the risk of postoperative ANFH, and those with preoperative traction had a lower risk of postoperative ANFH than those without. Based on these findings, clinicians may identify patients at a risk of postoperative ANFH in age, sex and surgical method subgroups, and take corresponding interventions to prevent and control the risk.

This study enrolled 427 patients, and focused on the associations of DDH classification and preoperative traction with the risk of postoperative ANFH as regards age at reduction, sex and surgical method. Nevertheless, several limitations should not be ignored when interpreting the findings. First, this study was a single-center retrospective study, which had a relatively poor sample representation and limited patient data. Global decreased enhancement of the femoral head, redislocation, and surgical treatment without femoral shaft shortening were found to be risk factors for postoperative ANFH (25–27). Since the retrospective nature of this study, some data cannot be obtained at present, and more factors need to be evaluated in the future. Second, the follow-up time was relatively short, whereas this follow-up time was sufficient to determine whether ANFH occurred. Third, since the surgery was not performed by the same surgeon, differences were inevitable, but all surgeons were qualified and experienced.

## Conclusion

For children aged 1–3 years, preoperative traction was associated with a significantly reduced risk of postoperative ANFH, and for children aged >3 years, DDH classification was positively associated with the risk of postoperative ANFH; among girls, those with a more severe DDH type had a significantly higher risk of postoperative ANFH, and preoperative traction was correlated with a significantly decreased risk of postoperative ANFH; regarding children undergoing open reduction, DDH classification was in a positive association with the risk of postoperative ANFH, and those with preoperative traction had a lower risk of postoperative ANFH than those without. Future investigations are needed to verify these findings.

## Data Availability

The raw data supporting the conclusions of this article will be made available by the authors, without undue reservation.

## References

[1] Wang Y. Current concepts in developmental dysplasia of the hip and total hip arthroplasty. Arthroplasty (London, England). (2019) 1(1):2. 10.1186/s42836-019-0004-6 35240757 PMC8787940

[2] Benjamin B Haddad FS. Management of limb length problems during total hip arthroplasty for patients with developmental dysplasia of the hip. Br J Hosp Med (Lond). (2020) 81(7):1–7. 10.12968/hmed.2019.0362 32730164

[3] Yang S Zusman N Lieberman E Goldstein RY. Developmental dysplasia of the hip. Pediatrics. (2019) 143(1):e20181147. 10.1542/peds.2018-1147 30587534

[4] Vaquero-Picado A González-Morán G Garay EG Moraleda L. Developmental dysplasia of the hip: update of management. EFORT Open Rev. (2019) 4(9):548–56. 10.1302/2058-5241.4.180019 31598333 PMC6771078

[5] Guerado E Caso E. The physiopathology of avascular necrosis of the femoral head: an update. Injury. (2016) 47(Suppl 6):S16–s26. 10.1016/S0020-1383(16)30835-X 28040082

[6] Palekar G. Hip preservation with autologous osteoblast cell-based treatment in osteonecrosis of the femoral head. Orthopedics. (2021) 44(2):e183–9. 10.3928/01477447-20201210-02 33316818

[7] Sibiński M Synder M Domzalski M Grzegorzewski A. Risk factors for avascular necrosis after closed hip reduction in developmental dysplasia of the hip. Ortop Traumatol Rehabil. (2004) 6(1):60–6. 17676009

[8] Schur MD Lee C Arkader A Catalano A Choi PD. Risk factors for avascular necrosis after closed reduction for developmental dysplasia of the hip. J Child Orthop. (2016) 10(3):185–92. 10.1007/s11832-016-0743-7 27177477 PMC4909658

[9] Cha SM Shin HD Shin BK. Long-term results of closed reduction for developmental dislocation of the hip in children of walking age under eighteen months old. Int Orthop. (2018) 42(1):175–82. 10.1007/s00264-017-3685-x 29130113

[10] Bozkurt C Sarikaya B Sipahioğlu S Çetin BV Bekin Sarikaya PZ Kaptan AY Evaluation of avascular necrosis risk factors after closed reduction for developmental dysplasia of the hip before walking age. J Pediatr Orthop B. (2022) 31(3):237–41. 10.1097/BPB.0000000000000846 34116555

[11] Segal LS Boal DK Borthwick L Clark MW Localio AR Schwentker EP. Avascular necrosis after treatment of DDH: the protective influence of the ossific nucleus. J Pediatr Orthop. (1999) 19(2):177–84. 10.1097/01241398-199903000-00008 10088684

[12] Wang YJ Yang F Wu QJ Pan SN Li LY. Association between open or closed reduction and avascular necrosis in developmental dysplasia of the hip: a PRISMA-compliant meta-analysis of observational studies. Medicine (Baltimore). (2016) 95(29):e4276. 10.1097/MD.0000000000004276 27442664 PMC5265781

[13] Gardner RO Bradley CS Howard A Narayanan UG Wedge JH Kelley SP. The incidence of avascular necrosis and the radiographic outcome following medial open reduction in children with developmental dysplasia of the hip: a systematic review. Bone Joint J. (2014) 96-b(2):279–86. 10.1302/0301-620X.96B2.32361 24493198

[14] Qiu M Chen M Sun H Li D Cai Z Zhang W Avascular necrosis under different treatment in children with developmental dysplasia of the hip: a network meta-analysis. J Pediatr Orthop B. (2022) 31(4):319–26. 10.1097/BPB.0000000000000932 34751178

[15] Kumar SJJCR. Congenital dysplasia and dislocation of the hip in children and adults. Clin Radiol. (1987) 39(3):277. 10.1016/S0009-9260(88)80531-2

[16] Narayanan U Mulpuri K Sankar WN Clarke NM Hosalkar H Price CT. Reliability of a new radiographic classification for developmental dysplasia of the hip. J Pediatr Orthop. (2015) 35(5):478–84. 10.1097/BPO.0000000000000318 25264556 PMC4484663

[17] Standefer KD Pierce WA Sucato DJ Kim HK. Detecting a disruption of blood flow to the femoral head after ischemic injury using 4 different techniques: a preliminary study. J Pediatr Orthop. (2012) 32(1):75–80. 10.1097/BPO.0b013e31823b1a90 22173392

[18] Bradley CS Perry DC Wedge JH Murnaghan ML Kelley SP. Avascular necrosis following closed reduction for treatment of developmental dysplasia of the hip: a systematic review. J Child Orthop. (2016) 10(6):627–32. 10.1007/s11832-016-0776-y 27812914 PMC5145826

[19] Li YQ Li M Guo YM Shen XT Mei HB Chen SY Traction does not decrease failure of reduction and femoral head avascular necrosis in patients aged 6-24 months with developmental dysplasia of the hip treated by closed reduction: a review of 385 patients and meta-analysis. J Pediatr Orthop B. (2019) 28(5):436–41. 10.1097/BPB.0000000000000586 30585878

[20] Farsetti P Efremov K Caterini A Marsiolo M De Maio F Ippolito E. The effectiveness of preliminary traction in the treatment of congenital dislocation of the hip. J Orthop Traumatol. (2021) 22(1):26. 10.1186/s10195-021-00586-8 34180020 PMC8236418

[21] Wu J Yuan Z Li J Zhu M Canavese F Xun F Does the size of the femoral head correlate with the incidence of avascular necrosis of the proximal femoral epiphysis in children with developmental dysplasia of the hip treated by closed reduction? J Child Orthop. (2020) 14(3):175–83. 10.1302/1863-2548.14.190176 32582384 PMC7302414

[22] Xu M Gao S Sun J Yang Y Song Y Han R Predictive values for the severity of avascular necrosis from the initial evaluation in closed reduction of developmental dysplasia of the hip. J Pediatr Orthop B. (2013) 22(3):179–83. 10.1097/BPB.0b013e32835f1f7a 23443144

[23] Cooper AP Doddabasappa SN Mulpuri K. Evidence-based management of developmental dysplasia of the hip. Orthop Clin North Am. (2014) 45(3):341–54. 10.1016/j.ocl.2014.03.005 24975762

[24] Bassett GS Barton KL Skaggs DL. Laser Doppler flowmetry during open reduction for developmental dysplasia of the hip. Clin Orthop Relat Res. (1997) 340:158–64. 10.1097/00003086-199707000-00020 9224251

[25] Cheon JE Kim JY Choi YH Kim WS Cho TJ Yoo WJ. MRI Risk factors for development of avascular necrosis after closed reduction of developmental dysplasia of the hip: predictive value of contrast-enhanced MRI. PLoS One. (2021) 16(3):e0248701. 10.1371/journal.pone.0248701 33735261 PMC7971487

[26] Pospischill R Weninger J Ganger R Altenhuber J Grill F. Does open reduction of the developmental dislocated hip increase the risk of osteonecrosis? Clin Orthop Relat Res. (2012) 470(1):250–60. 10.1007/s11999-011-1929-4 21643924 PMC3237975

[27] Domzalski M Synder M. Avascular necrosis after surgical treatment for development dysplasia of the hip. Int Orthop. (2004) 28(2):65–8. 10.1007/s00264-003-0522-1 15274235 PMC3474479

